# Evidence for risk extrapolation in decision making by tadpoles

**DOI:** 10.1038/srep43255

**Published:** 2017-02-23

**Authors:** Adam L. Crane, Maud C. O. Ferrari

**Affiliations:** 1 Department of Biology, University of Saskatchewan, 112 Science Place, Saskatoon, SK, S7N 5E2, Canada; 2 Department of Veterinary Biomedical Science, University of Saskatchewan, 52 Campus Drive, Saskatoon, SK, S7N 5B4, Canada

## Abstract

Through time, the activity patterns, morphology, and development of both predators and prey change, which in turn alter the relative vulnerability of prey to their coexisting predators. Recognizing these changes can thus allow prey to make optimal decisions by projecting risk trends into the future. We used tadpoles (*Lithobates sylvaticus*) to test the hypothesis that tadpoles can extrapolate information about predation risk from past information. We exposed tadpoles to an odour that represented either a temporally consistent risk or an increasing risk. When tested for their response to the odour, the initial antipredator behaviour of tadpoles did not differ, appearing to approach the limit of their maximum response, but exposure to increasing risk induced longer retention of these responses. When repeating the experiment using lower risk levels, heightened responses occurred for tadpoles exposed to increasing risk, and the strongest responses were exhibited by those that received an abrupt increase compared to a steady increase. Our results indicate that tadpoles can assess risk trends through time and adjust their antipredator responses in a way consistent with an extrapolated trend. This is a sophisticated method for prey to avoid threats that are becoming more (or less) dangerous over part of their lifespan.

A major challenge for prey is navigating a landscape of predation risk that fluctuates across temporal and spatial scales^1^,^2^. Predators come and go according to diel and annual activity patterns. Prey, too, experience changes that can profoundly affect their risk landscape^3^,^4^,^5^,^6^. For instance, many prey species undergo major habitat shifts during migrations or when transitioning through life-history stages, sometimes moving from aquatic to terrestrial habitats or vice versa (e.g. refs 7,8, 9, 10, 11). As these changes occur, predators from the former habitat will become non-threats while predators in the new habitat will become dangerous (e.g. refs 12 and 13).

Young prey undergo major changes in growth and development (e.g. refs 14, 15, 16) that can also modify their vulnerability to specific predators. For instance, larval anurans (tadpoles) face a diverse community of predators upon hatching. Over time, they outgrow many of these predators (e.g., gape-limited invertebrates and salamander larvae)^17^,^18^,^19^. At the same time, however, they become more vulnerable to other predators (e.g., large invertebrates and birds)^20^,^21^, as they become more conspicous and energetically profitable.

Prey must avoid predation in order to survive, but antipredator responses require time and energy that are important for other critical activities such as foraging, reproduction and competition^22^,^23^,^24^. Thus, correclty recognizing and responding to predation risk is essential for maximizing benefits relative to costs^25^,^26^. Some prey species respond to risk by altering their morphology and the timing of life-history switches, and antipredator behaviours are common, being relatively low in cost and rapidly triggered^27^,^28^,^29^. The intensity of antipredator responses are fine-tuned to match the perceived risk of predation, where larger threats elicit stronger antipredator responses^30^,^31^,^32^.

Learning about predator cues, either from direct experience or from social information, is an important way to avoid future predator attacks^33^,^34^. However, in changing environments learned information can quickly become outdated, and thus prey should continuously use new information about predation risk to update what they have learned previously^35^,^36^,^37^. Therefore, prey that are capable of learning patterns of risk can make better antipredator decisions^38^,^39^,^40^. When new information conflicts with prior assessments, prey can disregard either the new or prior information, or they can use both sources of information to better estimate the future threat^36^,^41^.

A few theoretical models predict how animals should optimally use past and present information^6^,^27^,^28^. In relatively constant environments, all information should contribute equally to a decision, and thus, animals should average prior information^42^,^43^. However, in highly variable environments where information changes rapidly, prey might benefit from relying on the most recent and thus more accurate, information^42^,^44^,^45^. These models were developed in the context of foraging and patch use, but little is known about whether the same decision-making rules apply for antipredator decisions, which we sought to explore here.

We used larval wood frogs (*Lithobates sylvaticus*) to assess how patterns of risk affect future responses to that risk. We used wood frogs because of the extensive amount of information known about their antipredator behaviour and cognition. Wood frogs face intense predation pressure that results in only a tiny percentage of individuals surviving to adulthood^46^, and thus, there is strong selection for fine-tuned, antipredator strategies^27^,^47^. Wood frog tadpoles recognize risk in their environment from cues released by injured conspecifics (hereafter ‘alarm cues’)^48^,^49^. To nearby individuals, these cues indicate that a predator attack has occurred. Tadpoles typically respond by decreasing their activity which limits detection by predators^50^,^51^. For wood frog tadpoles, one way to learn about a predator is by encountering the predator’s cues alongside alarm cues (a pairing often referred to as ‘conditioning’)^40^,^50^. Moreover, the concentration of the cues can mediate the intensity of the threat, with high and low concentrations of alarm cues resulting in tadpoles learning to recognize the predator as a high or low threat, respectively^52^. A few studies that have conditioned tadpoles to recognize a novel predator odour did so by conditioning tadpoles multiple times^40^,^53^,^54^. In these studies, when new information was consistent with prior information, tadpoles showed similar learned responses after being conditioned either once or multiple times. However, individuals that were conditioned multiple times showed a longer retention of their learned responses^53^. In another study, where current information (high risk) conflicted with past information (low risk), tadpoles responded with a higher level of antipredator behaviour compared to individuals that were conditioned only with the high risk level^54^.

Our goal here was to better understand how tadpoles use prior trends in risk to adjust their future antipredator responses. We propose that exposure to trends in risk could cause tadpoles to anticipate that the trend will continue. Thus, tadpoles might extrapolate their responses beyond the previous risk levels. For instance, prey should display stronger antipredator responses toward a predator cue that changes from being low risk to a high risk, compared to a predator cue that consistently represents high risk.

While making predictions for outcomes based on different decision-making rules (see Table 1), we sought evidence for the extrapolation hypothesis. In two experiemnts with different overall risk levels, we exposed tadpoles to various risk patterns from a novel predator over a 4-day period. Some tadpoles were exposed to a predator cue that represented either a consistently high threat (high-high-high), or a non-threat (nil-nil-nil risk control). Other tadpoles were exposed to a predator cue representing an increasing threat, starting as a low threat and ending at a risk level that matched that of the consistantly high-risk group.

To further understand how tadpoles would incorporate increasing risk scenarios through time, we introduced two treatments: a steady increase (low-medium-high) or an abrupt increase (low-sham-high) (Table 1). We predicted that if tadpoles averaged the information they experienced through time, the exposure to consistent high risk would elicit stronger responses than would any other treatment. Alternatively, if tadpoles relied only on their most recent experience, individuals receiving the same level of risk on their final conditioning (high) would exhibit similar responses regardless of prior risk levels. However, if tadpoles extrapolated risk levels, exposure to both increasing-risk treatments should result in responses that are even stronger in intensity than those of individuals exposed to consistent high risk. While both increasing-risk groups represented a similar rate of increase in risk (from low to high over the same amount of time), one group (low-medium-high) was more informed and hence less ‘mismatched’ based on the previous information. In other words, the discrepancy between the two most recent assessments was larger for the abrupt-increase group. If tadpoles did not extrapolate risk levels, we should see no difference between these two groups. However, if the mismatch in risk influenced their responses, we hypothesized that the abrupt-increase group would show a stronger response, and a response maintained for a longer duration.

## Results

### Experiment 1

**Figure 1:**
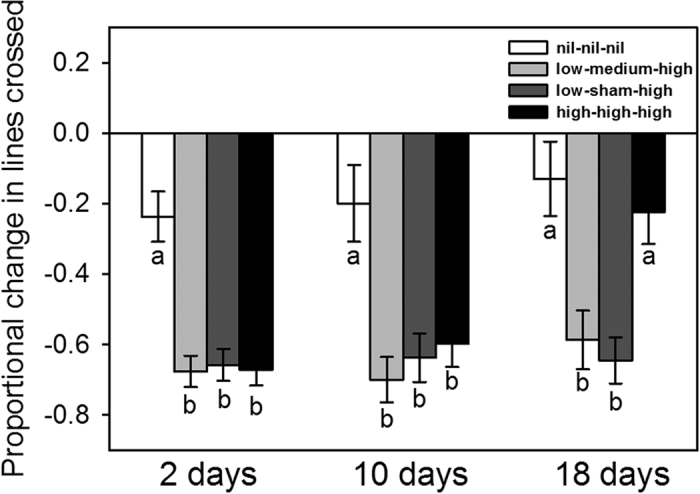
Mean (±SE) proportional change in lines crossed for tadpoles conditioned with novel predator odour paired with various concentrations of alarm cues three times over four days and then tested either 2, 10, or 18 days post-conditioning. Letters indicate significant differences found with Tukey post-hoc tests.

Following the injection of predator odour, the proportional changes in movement of tadpoles in each treatment depended on the time since conditioning (treatment × time since conditioning *F*_6, 313_ = 3.5, *P* = 0.002; nest *F*_36, 284_ = 1.2, *P* = 0.21; Fig. 1). Post-hoc analyses revealed that tadpoles in the alarm cue treatments (i.e., consistent high risk, steady increase, and abrupt increase) learned to decrease movement in response to the predator odour. At 2 and 10 days post-conditioning, their learned responses were similar (2 days: treatment *F*_3, 36_ = 15.1, *P* < 0.001; nest *F*_36, 115_ = 1.1, *P* = 0.38; 10 days: treatment *F*_3, 29_ = 11.8, *P* < 0.001; nest *F*_36, 52_ = 0.66, *P* = 0.91), but at 18 days, only tadpoles in the increasing-risk treatments (steady and abrupt) continued to display learned responses (treatment *F*_3, 11_ = 13.7, *P* < 0.001; nest *F*_31, 50_ = 0.7, *P* = 0.87). There was no difference between the intensity of responses of individuals in the steady-increase treatment and the abrupt-increase treatment (Fig. 1).

### Experiment 2

**Figure 2:**
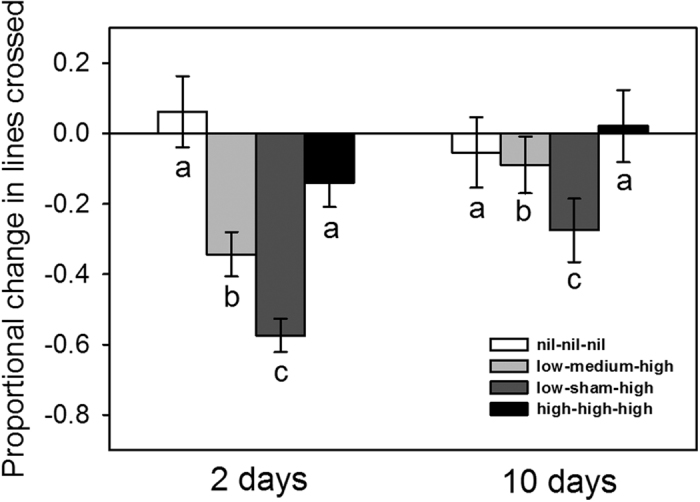
Mean (±SE) proportional change in lines crossed for tadpoles conditioned with novel predator odour paired with various concentrations of alarm cues three times over five days and then tested either 2 or 10 days post-conditioning. Letters represent significant main effects.

By repeating experiment 1 using lower risk levels, we found a different pattern, one that supported the risk extrapolation hypothesis. The learned responses of tadpoles differed among the treatments (*F*_3, 34_ = 12.7, *P* < 0.001; Fig. 2) with only tadpoles in the increasing-risk treatments displaying significantly stronger antipredator responses than those in the control (steady increase vs. no risk: *P* = 0.016; abrupt increase vs. no risk: *P* < 0.001; consistent high risk vs. no risk: *P* = 0.62). Moreover, individuals that received an abrupt-increase in risk exhibited significantly stronger responses than those in the steady-increase treatment (*P* = 0.041), supporting the hypothesis that the larger mismatch between past risk assessments spurred the more intense response. At 10 days, the overall learned responses were weaker (*F*_1, 283_ = 5.4, *P* = 0.021), and differences among treatments appeared mitigated although there was not a significant interaction between the treatment and time since testing (interaction *F*_3, 280_ = 2.0, *P* = 0.12; nest *F*_36, 258_ = 0.9, *P* = 0.56), so no further testing was conducted. All data are available as Supplementary Information.

## Discussion

We found strong support that tadpoles estimated future risk by using increasing risk trends. In experiment 2, tadpoles clearly exhibited stronger antipredator responses after being conditioned with increasing alarm cue concentrations compared to those conditioned with consistent high risk (Fig. 2). This is a sophisticated strategy for estimating risk outside the range of prior risk, and to our knowledge, this is the first study to formally test the ability of prey animals to display behavioural responses consistent with extrapolation trends.

While tadpoles from both increasing-risk treatments showed extrapolated responses, the most intense responses were exhibited by tadpoles exposed to the abrupt increase in risk (Fig. 2). This indicates that extrapolation was not based on the rate of increase because both increasing-risk treatments had the same change in risk over the same amount of time. Instead, the larger extrapolated response appeared to result from the larger discrepancy with past experience, which may have indicated to tadpoles that their prior assessment was more incorrect in the context of the new information.We believe the intensity of tadpoles’ extrapolated responses are based on adding this past discrepancy to their baseline threat-sensitive responses.

Unlike experiment 2, risk extrapolation was absent in experiment 1 when alarm cue concentrations were higher overall (Fig. 1). Presumably tadpoles were approaching the limit of their functional plasticity (i.e., their maximal response in the context of behavioural trade-offs), which likely has a hormonal basis^55^. This outcome matches several other studies in this tadpole system, where using the same testing methodology resulted in the most intense reductions in movement of ~60% (e.g. refs 50,53 and 56). In contrast, when concentrations were lower in experiment 2, tadpoles in the consistent-risk treatment reduced movement by only ~14% which is substantially less than tadpoles at similar risk levels in other studies^54^,^56^. However, the tadpoles in those studies were two months younger and from a different population, both of which can affect behaviour (e.g. refs 57,58, 59, 60).

We observed a longer retention of the learned response among tadpoles that experienced increasing risk. In experiment 1, both steady and abrupt increases resulted in fully retained responses for 18 days (Fig. 1). However, retention was much shorter after exposure to risk at lower concentrations (Fig. 2). In this case, tadpoles in neither increasing-risk treatment maintained their response intensity after 10 days (i.e., the responses weakened compared to those at two days). These reductions in intensity occurred at similar rates (Fig. 2), suggesting an abrupt increase in risk results in longer retention of learned responses only because the speed of waining is similar. The loss of learned responses may not be the result of forgetting the information. In previous studies, tadpoles that lost their conditioned responses in one context were then affected by their earlier conditioning in subsequent contexts^54^,^61^. Thus, the learned information was not forgotten, but rather it was simply not being used at that time. The weakening of learned responses over time may be partly the result of an updating process where responses weaken according to the irregularity/absence of predator encounter^62^,^63^.

A potential alternative hypothesis for our results in experiment 2 is habituation (i.e., the lessening of a behavioural response due to repeated stimulation^64^). Tadpoles received repeated conditionings, and individuals receiving more (3 vs. 2), or at higher alarm cue concentrations, potentially could have habituated more. Several studies have examined whether repeated conditionings can lead to either habituation or to more intense learned responses, with most finding enhanced effects (e.g., Arctic charr, *Salvelinus alpinus*^65^; rhesus monkeys, *Macaca mulata*; New Zealand robins, *Petrocia ausralis*^66^,^67^), although we are aware of two studies showing a weakening effect (chinook salmon, *Oncorhynchus tshawytscha*^68^; tammar wallabies, *Macropus eugenii*^69^). Previous studies with wood frog tadpoles, however, have consistently found no habituation to multiple conditionings (up to 4 more conditionings than in this study)^40^,^53^,^54^,^70^. Instead, multiple conditionings led to longer retention of learned fright responses. Moreover, our data from experiment 1 do not provide evidence of habituation, as tadpoles from all risk treatments appeared to approach the limit of their functional response. Thus, we can provisionally rule out habituation to risk by tadpoles in our study.

The time frame of the change in risk likely influences the extrapolated responses. In experiment 2, tadpoles exhibited an extrapolated response to the predator odour two days after risk had increased over a 4-day period. In previous work, where increasing risk occurred over a longer time period (two weeks), the extrapolated response occurred only at a later time point (11 vs. 1 day)^54^. Together, these studies suggest that tadpoles use the time frame of changing risk to estimate the appropriate timing of their extrapolated responses. More work, however, is needed to assess the trajectory of this response. If tadpoles are using a linear trajectory, the intensity of their responses would incrementally increase through time along the slope of the past increase. Alternatively, tadpoles may use a nonlinear trajectory such as a staircase trajectory. In this case, new information about increased risk levels would cause an immediate uptick in the intensity of their response, which would then be maintained over a time frame that reflects the time between the two most recent assessments, where changes over longer periods would result in longer retention of the extrapolated response.

Extrapolating from risk trends should be a beneficial strategy for making correct antipredator decisions in environments where predators become more or less dangerous over time, such as when prey are undergoing developmental changes. Our results are consistent with the hypothesis that tadpoles can extrapolate from prior risk trends, providing new insight into their surprisingly sophisticated decision making.

## Methods

### Ethical statement

This study was conducted in accordance with the guidelines of the Canadian Council on Animal Care, and was approved by the University of Saskatchewan’s Committee on Animal Care (protocols 20060014 and 20100113).

### Animal collection, maintenance, and cues

In May 2014, we collected seven clutches of wood frog eggs from five roadside ponds in central Saskatchewan the morning after they had been laid. We equally divided the eggs into twelve outdoor plastic pools (42 cm height, 48 cm diameter) filled with 65 L of filtered water. After hatching, tadpoles were fed alfalfa pellets and algae with a 30% water change every 2 d. At the time of the experiment (late August), tadpoles were free-swimming and began to quickly develop their posterior limbs (developmental stage 30–38 according to Gosner^71^), one of several periods where their vulnerability to specific predators may change. All procedures were conducted outdoors.

Tadpole alarm cues are a reliable indicator of predation risk that is recognized innately^72^ in a threat-sensitve manner^50^,^73^. We prepared alarm cues by rapidly euthanizing tadpoles with a blow to the head and crushing individuals with a mortar and pestle This homogenized mix of alarm cues from several tadpoles was then diluted in different volumes of water to produce different concentrations for each treatment (see below), and hence different levels of risk. The solution was filtered through cotton wool to remove any solid particles.

Four tiger salamanders (*Ambystoma tigrinum*) from a stock colony at the University of Saskatchewan were used as novel predators. Salamanders (snout-vent length: 10–11 cm) were housed individually in plastic holding containers (30 × 16 × 12 cm) with water and were fed earthworms. We obtained their odour by keeping salamanders individually in 1 L of clean water for 24 h. The water from the four individuals was mixed and frozen in 1 L bags. Numerus studies in this system have shown that tadpoles do not recognize these cues innately but learn them as risky from a pairing with alarm cues (e.g. refs 74 and 75).

### Experiment 1

In late July 2014, we used 40 1-L plastic containers (10 × 10 × 9 cm), each housing eight tadpoles during conditioning. Tadpoles had 24 h to acclimate before receiving the first of three exposures over 4 d with 48 h between each exposure. Consistent high risk involved three conditionings of predator odour paired with a high concentration of alarm cues (high-high-high), whereas the no-risk control was three exposures to predator odour + water (nil-nil-nil). For the increasing risk, predator odour was paired initially with a lower concentration of alarm cues but then with higher concentrations of alarm cues at subsequent exposures, the final of which matched the high concentration of the consistent high-risk (low-medium-high; low-sham-high). The high risk solution was 1.5 crushed tadpoles per 10 mL, while the medium risk and low risk solutions were 1 tadpoles/10 mL and 0.5 tadpoles/10 mL respectively. After being added into the 1-L containers, these solutions closely match the ‘high’, ‘medium’ and ‘low’ concentrations used in previous studies to elicit high, medium, and low antipredator responses in this species e.g. refs 50 and 56. For the risk exposures, we gently injected 10 mL of predator odour paired with one of the 10-mL alarm-cue solutions because wood frog tadpoles do not innately recognize this odour as risky. A no-risk exposure consisted of an injection of predator odour (10 mL) +blank water (10 mL), and the sham exposure was an injection of 20 mL of water. We did not use predator odour in the sham exposure because we did not want tadpoles to experience the predator without information regarding risk. Water was changed one hour following each exposure.

We tested tadpoles individually for their antipredator responses at either 2, 10, or 18 days post-conditioning when they were 18–28 mm in total length at Gosner stages 30–36. First, tadpoles were placed into arenas (0.5 L plastic cups filled with 480 mL of water) and were allowed to acclimate for 1 h. A dividing line on the arena allowed us to quantify movement. We conducted 4-min observation periods before and after injecting 5 mL of predator odour. The number of lines crossed (defined by the entire body crossing the line) was recorded for both the pre- and post-stimulus periods. We calculated the proportional change [(post-pre)/pre] in lines crossed to account for any variation in pre-stimulus movement. Sample sizes were >20 per group. Tadpoles were not tested more than once.

### Experiment 2: repeating experiment 1 using lower risk levels

Based on the results from Experiment 1, we suspected that the high-risk concentrations could result in tadpoles displaying behavioural responses that approached the upper limit of their maximum response threshold. In other words, while tadpoles may differ in their perceived risk associated with the predator, the asymptotic nature of antipredator response for high risk would hide this difference behaviourally. Therefore, we conducted a second experiment (late August 2014) at lower risk levels to increase our chance of seeing behavioural differences between groups. We reduced alarm-cue concentrations by 50% [0.75 tadpoles/10 mL (high), 0.5 tadpoles/10 mL (medium), and 0.25 TP/10 mL (low)], closer to their response threshold^56^,^76^, and patterned the treatments as in experiment 1: consistent high risk (high-high-high), steady increase (low-medium-high), abrupt increase (low-sham-high), and no risk (nil-nil-nil). At the time of testing, tadpoles were 20–30 mm in total length at Gosner stages 32–38. Sample sizes were >30 per group. All other conditioning and testing procedures were conducted as in experiment 1.

### Statistical analyses

We used 2-way nested ANOVAs to analyze the data obtained from each experiment. The risk treatment (no risk, consistent high risk, steady increase or abrupt increase) and the time since conditioning (2, 10, or 18 days) were fixed factors and the conditioning container was the nested factor to ensure that the container, and not the tadpole, was our unit of replication (tadpoles conditioned in the same container are not independent). To better interpret interactions, we performed post-hoc 1-way nested ANOVAs with Tukey comparisons for each testing time. We used α = 0.05, and analyses were conducted in SPSS 21.

## Additional Information

**How to cite this article**: Crane, A. L. and Ferrari, M. C. O. Evidence for risk extrapolation in decision making by tadpoles. *Sci. Rep.* **7**, 43255; doi: 10.1038/srep43255 (2017).

**Publisher's note:** Springer Nature remains neutral with regard to jurisdictional claims in published maps and institutional affiliations.

## Supplementary Material

Supplementary Information

## Figures and Tables

**Table 1:** Predictions of behavioural outcomes for potential strategies used by prey to predict the change in risk through time based on prior information.

Treatment	Potential strategies:		
	use recent	average	extrapolate
consistent high risk: *high-high-high*	high	high	high
no risk: *nil-nil-nil*	nil	nil	nil
steady increase: *low-medium-high*	high	medium	>high
abrupt increase: *low-sham-high*	high	medium	>high

Potential strategies include using only the most recent information, averaging all information through time, and extrapolating from prior risk patterns. High/medium/>high refers to the intensity of response displayed by the prey when exposed to the predator cues.
